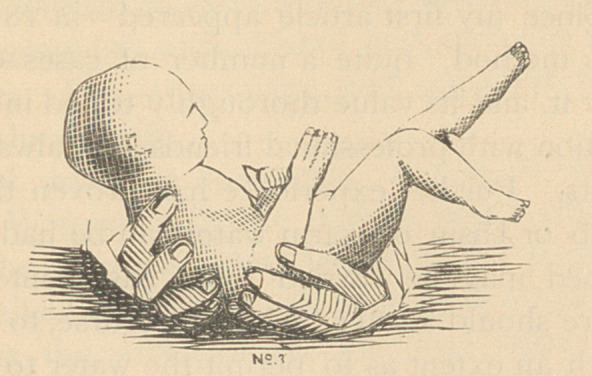# A “Speedy Method” in Asphyxia

**Published:** 1880-01

**Authors:** Harvey L. Byrd

**Affiliations:** Baltimore, Md.; Formerly Professor in the Savannah Medical College, and Oglethorpe Medical College, of Georgia; late Professor in Washington University, and College of Physicians and Surgeons of Md., etc.; 147 Edmondson Avenue


### ARTICLE III.

### A "SPEEDY METHOD" IN ASPHYXIA.

BY HARVEY L. BYRD, M. D., ETC., BALTIMORE, MD.

(Formerly Professor in the Savannah Medical College, and Oglethorpe Medical College, of Georgia; late Professor in Washington University, and College of Physicians and Surgeons of Md., etc.)

It is hardly necessary in an article like this to urge professional attention to the vast importance, in a medico-legal point of view, of establishing, even for a moment or two, the vitally necessary function of respiration in the newly-born infant. And whether a child be considered a "living soul" or not ere breathing occurs, all will agree that humanity calls loudly for prompt and efficient action at the hand of the officiating accoucheur for the development of the respiratory process in all cases where children emerge into the world in an asphyxiated condition.

Asphyxia is liable to be produced by a variety of causes; and every abnormal state or unusual circumstance occurring during labor likely to result in this critical and dangerous condition to the infant, should be carefully weighed by the attending obstetrician, so as to be prepared to promptly meet the emergency, should it arise. On the announcement to the profession of Dr. Marshall Hall's "ready method" in asphyxia, some years ago, and its practical application in a few cases, the writer thought there was very little, if any, further addition necessary to the list of remedial agents in the asphyxia of newly-born infants. Later experience, however, with this and subsequently published "methods," proved that, occasionally at least, all the then known appliances were futile, and further knowledge required to secure success in the management of this very dangerous condition of the infant. The following "method," it is believed, will be found a highly valuable, if not the most important, addition to our list of appliances in the asphyxia of children, and also for the relief of that condition in the adult, when properly manipulated. The procedure is easy of accomplishment, and requires no preliminary arrangement or preparation for its application, but may be put into execution the moment the condition of the child may demand it. It is as follows: Bring the *ulnar* sides of the hands near together, with the palmar surfaces looking vertically, and place them beneath the back of the infant, so that the extended thumbs may aid, as far as possible, in sustaining the vertex and inferior extremities; then, keeping the *ulnar borders* near together, so as to

No. 1

No. 2.

form a fulcrum, the radial borders or sides are simultaneously depressed to as great extent as practical—say forty five degrees—*below* the horizontal line, and then gradually pronated or elevated to as many degrees *above* that line, thus facilitating the escape of air drawn into the lungs during the downward movement of the head and chest. Or the hands are placed as at first, and passed *beneath* the body of the child—on its back—and the superior and inferior extremities furthest from the operator seized, one by each hand, near the trunk—the *ulnar borders* of the hands and wrists forming the fulcrum—the head of the child being kept at a proper axis with the movements of the chest by the hands of an assistant; and the depression and elevation of the head and lower extremities proceeded with as already described. These alternate depressions and elevations of the two extremities, performed in a regular and gentle manner, and repeated at proper intervals, seldom fail in establishing respiration where it is

No. 3

possible of accomplishment. The occasional dashing of cold water on the epigastrium during the *descent* of the head and chest will hasten respiration where the first few movements fail in its establishment. It is important that the head be kept, as far as practicable, from too much *lateral* movement, and not permitted to depart considerably from its *antero-posterior* axis with the vertebral column during the continuance of the process. To this end, in a critical case, the hands of an assistant may be brought into requisition. The importance of these remarks will be apparent to intelligent readers on a moment's reflection. No impediment should be *permitted* in the way of *free entrance* of air into the lungs during the *downward* movement of the head; and it is *scarcely less important* that no *obstruction* should oppose the escape of air during the *upward* movement of the head and chest. The philosophy of the above-described movements will be easily comprehended by a glance at the accompanying wood-cuts. A nurse or other intelligent attendant can be made to understand the movements, so as to continue them should the condition of the mother demand the attention of the accoucheur. These movements will apply to the treatment of asphyxiated persons of *any age*, as has been practically demonstrated in several cases since the publication of my first article on the subject. Asphyxia from drowning has been promptly overcome in three instances since the question was asked in a former communication—on theoretical grounds—whether it would not act more promptly than *any other* method. It has been found that the "movements" are easily practiced when the body is taken from the water and placed on its back across a barrel, trunk of a fallen tree, or other substance. Two persons can thus depress and elevate the extremities as often as necessary to expand and exhaust the air in the lungs, as in normal respiration. The epiglottis acts in these movements in a manner similar to the valve of an ordinary bellows when being used. Since my first article appeared—in 1870—on the value of the "speedy method," quite a number of cases of asphyxia have been treated by it, and its value thoroughly tested in my own practice, and in consultation with professional friends, and always with the most gratifying results. Further experience has proven that where a sufficiently large tub or basin of warm water can be had, the movements already described may be carried more easily into operation than otherwise. Care should always be had, of course, to avoid depressing the head to such an extent as to permit the water to enter the mouth or nostrils of the child during the operation. In practicing the "method" *in the water* the head need not at any time be elevated above the surface—the body of the child alone being elevated or depressed—for the inflation and exhaustion of the air in and from the lungs. A number of cases could be recorded in favor of the method since the previous reports were made, but they are deemed unnecessary at this time. The object of this communication is to give greater publicity to a most important remedial procedure, and we may here repeat what has already been proven to be true by many practitioners, "that those who may have occasion to employ this method will agree with us that in the asphyxia of children it is the remedy *par excellence*."

147 EDMONDSON AVENUE.

## Figures and Tables

**No 1 f1:**
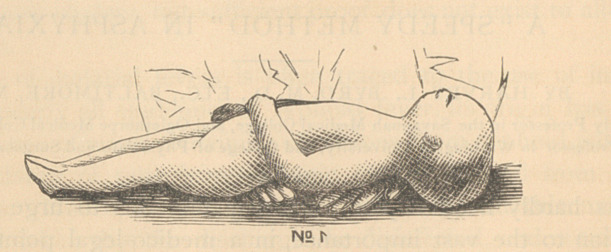


**No 2. f2:**
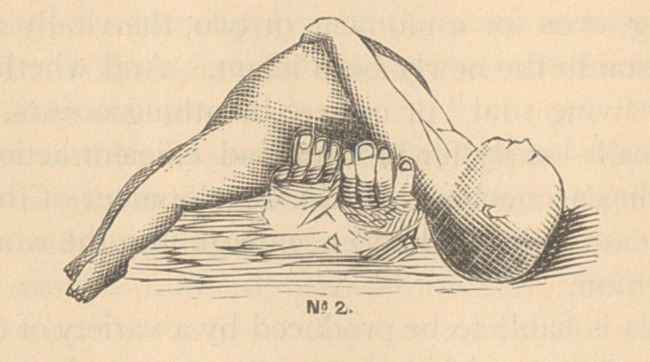


**No 3. f3:**